# Efficacy of Immune Checkpoint Inhibitors in Upper Tract Urothelial Carcinomas: Current Knowledge and Future Directions

**DOI:** 10.3390/cancers13174341

**Published:** 2021-08-27

**Authors:** Jonathan Thouvenin, Nieves Martínez Chanzá, Omar Alhalabi, Hervé Lang, Nizar M. Tannir, Philippe Barthélémy, Gabriel G. Malouf

**Affiliations:** 1 Oncology Department, Institut de Cancérologie Strasbourg (ICANS), 67200 Strasbourg, France; p.barthelemy@icans.eu; 2 Oncology Department, Institut Jules Bordet, 1000 Bruxelles, Belgium; Nieves.martinez-chanza@bordet.be; 3 Genitourinary Medical Oncology Department, The University of Texas MD Anderson Cancer Center (MDACC), Houston, TX 77030, USA; OAlhalabi@mdanderson.org (O.A.); NTannir@mdanderson.org (N.M.T.); 4 Urology Department, Strasbourg University Hospital, Nouvel Hôpital Civil, 67000 Strasbourg, France; herve.lang@chru-strasbourg.fr

**Keywords:** immune checkpoint inhibitors, immunotherapy, upper tract urothelial carcinoma, UTUC, genetic, epigenetic

## Abstract

**Simple Summary:**

Upper tract urothelial carcinoma (UTUC) represents 5 to 10% of urothelial carcinoma. Their mutational profile is different as compared to bladder urothelial carcinoma (UC). While immune checkpoint inhibitors are now part of the therapeutic landscape of urothelial carcinoma, data concerning their use in UTUC patient’s treatment remain scarce. The aim of this review is to summarize the available evidence and the biological rationale of using immune checkpoint inhibitors in high-grade UTUC. We reviewed the latest molecular characterization data and proposed an insight for future therapeutic strategies based on molecular alteration profiles.

**Abstract:**

Upper tract urothelial carcinoma (UTUC) represents a rare and aggressive malignancy arising from the renal pelvis or ureter. It can develop sporadically or have a hereditary origin, such as Lynch syndrome, caused by DNA mismatch repair deficiency, leading to microsatellite instability phenotype. According to molecular characterization studies, UTUC presents different mutational profiles as compared to urinary bladder urothelial carcinomas. In particular, it has been reported that UTUC harbored a higher level of *FGFR3* alterations associated with a T-cell depleted immune microenvironment. The therapeutic landscape in urothelial carcinoma is rapidly evolving, with immune checkpoint inhibitors forming part of the standard of care. A greater understanding of the molecular alterations and immune microenvironment leads to the development of new treatment combinations and targeted therapy. This review summarizes the available evidence concerning the use of immune checkpoint inhibitors and the biological rationale underlying their use in high-grade UTUC.

## 1. Introduction

Urothelial carcinoma (UC) represents the fourth most common malignancy worldwide, with an urgent need for tailored approaches in the management of the metastatic disease [1]. Depending on the level of muscle invasion seen on the pathological exam, UC is divided into muscle-invasive (MI) and non-muscle invasive (NMI) disease. MIUC of the bladder represents 25% of tumors [2] as compared to 60% in upper tract urothelial carcinoma (UTUC), explaining their increased aggressiveness [3,4,5,6]. The 5-year extravesical recurrence and overall survival rates are 28% and 23% for UTUC and bladder UC, respectively [7]. While bladder origin represents 90–95% of UCs, UTUC is less common. It represents 5–10% of UCs and can arise within the renal pelvis or ureter, which are derived from a different embryologic origin as compared to the bladder [1,8].

There is a strong relationship between UC of the bladder and UTUC since approximately 50% of patients with UTUC will have urinary bladder urothelial carcinomas either at presentation or subsequently, justifying the need to perform annual cystoscopy in the follow-up of these patients [1].

For high-risk localized disease, nephroureterectomy along with peri-operative chemotherapy is the standard of care management approach [1]. In the metastatic setting, platinum-based chemotherapy regimen remains the first-line recommended treatment [1]. However, there is a growing body of evidence concerning the use of immune checkpoint inhibitors (ICI) in the treatment of urothelial carcinoma [1,9] with the approval of several compounds in the first and second-line settings of advanced UCs. However, given their rarity, patients with UTUC represent a minority of patients included in clinical trials, and there is a paucity of data concerning ICI use in this setting.

UTUC has a different behavior as compared to bladder UC [10], and while molecular alterations of urothelial bladder carcinoma have been widely studied by The Cancer Genome Atlas, data about such alterations in UTUC remain scarce [4,11,12]. However, the novel molecular insights provided by these studies led to a better understanding of this aggressive disease and provided a rationale for new therapeutic approaches.

This review summarizes the available literature regarding the use of ICIs and the biological rationale underlying their use in high-grade urothelial upper tract carcinoma management.

## 2. The Molecular Landscape of UTUC

Despite histological similarities, UTUC displays different molecular and genetic features as compared to bladder UC [11,13,14,15]. Bladder UC was classified into 5 molecular subtypes, according to the TCGA: luminal-papillary, luminal-infiltrated, luminal, basal/squamous, and neuronal [14,16]. However, UTUC was not included in this analysis.

In the past few years, several studies have focused on the molecular characterization of UTUC using next-generation sequencing [4,5,6,11,12,13,17,18,19,20,21,22,23,24]. The most frequently mutated genes were *FGFR3* (40–80%), *KMT2D* (35–56%), *KMT2A* (32–47%), and *TP53* (18–26%) [4,5,11,13,22]. Other common mutations involving oncogenes or tumor suppressor genes, such as *HRAS*, *NRAS*, *KRAS*, *ARID1A*, *PIK3CA* and *CDKN2A* were also reported in these studies. Alteration of *TP53/MDM2* was associated with more aggressive disease and worse outcomes, whereas *FGFR3* alterations were linked to a better prognosis [13,20]. Comparable mutation rates were seen in UTUC and bladder UC, with a strong APOBEC signature, but *FGFR3*, chromatin remodeling gene such as *KMT2D*, and *CDKN2A* were mutated at a higher frequency in UTUC compared in bladder UC [4]. Conversely, the frequency of *TP53* mutations was lower. However, the associations between these somatic alterations and response to immunotherapy in UTUC are unclear. This is, for instance, the case for *CDKN2A* loss, which has been shown to be associated with decreased response to atezolizumab in bladder UC [25].

UTUC is represented in the spectrum of Lynch syndrome, an autosomal-dominant familial cancer syndrome caused by germline mutations in the DNA mismatch repair (MMR) genes *MLH1*, *MSH2*, *MSH6*, or *PMS2* [26,27,28]. It is particularly associated with *MSH2* mutations, with such mutations found in approximately 70% of UC integrated in Lynch syndrome [26,27,29,30]. Loss of function in the MMR system, either caused by an inherited mutation or a sporadic event, results in microsatellite instability (MSI) throughout the genome [31]. MSI have been found between 3.9% to 20% in UTUC as compared to <1% in UBC [32,33]. High MSI is correlated with a better prognosis, particularly in patients younger than 71 years old with T2-T3N0M0 tumors [34]. Furthermore, high MSI is associated with a higher tumor mutational burden (TMB) and correlated to a higher response rate with ICI treatment [35,36]. Recent retrospective data in advanced high MSI UTUC patients treated with immune checkpoint inhibitors have demonstrated excellent clinical activity [28].

Using a combination of whole-exome sequencing and RNA sequencing, some studies described biological differences between UTUC and bladder UC [11]. Four molecular subgroups of UTUC were defined through RNA-sequencing by Moss et al. [4]. Comparison to the TCGA dataset has revealed that cluster 1 was similar to luminal-subtype, cluster 2 was close to basal subtype, and cluster 4 demonstrated a high frequency of upregulated immune checkpoint related genes [4]. Moreover, UTUC is found to be predominantly luminal-like tumors, and, more precisely, luminal-papillary and T-cell depleted as compared to bladder UC [11,14].

**Figure 1:**
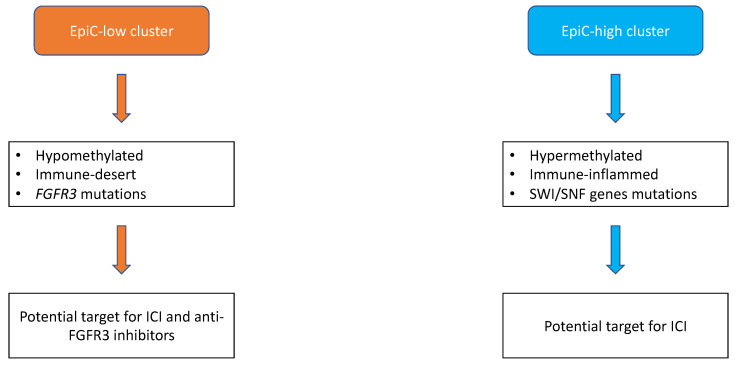
Proposal of molecular subtypes classification of upper-tract urothelial carcinomas adapted from Su et al. [12]. Broadly, upper-tract urothelial carcinomas can be divided into two subtypes, namely EpiC-high and EpiC-low. Epic-low subtype is hypomethylated, immune-desert, and characterized by *FGFR3* somatic mutations with potential efficacy of the combination of FGFR3 immunotherapy and immune checkpoint inhibitors (ICI). Conversely, EpiC-high subtype is hypermethylated, immune-inflamed, and enriched with somatic mutations of SWI/SNF genes with potential benefit for ICI.

Recently, we reported the UTUC methylation profiles and identified 2 epigenetic subtypes, namely EpiC-low and EpiC-high. While the earlier one was hypomethylated, immune depleted, and enriched for *FGFR3* mutations, the latter was hypermethylated, immune infiltrated, and associated with SWI/SNF genes somatic mutations [12] (Figure 1). Moreover, we identified for the first time a high rate of mutations in the ZFP36 family in almost one-quarter of UTUC. Further mechanistic studies showed that ZFP36L1 loss of function experiments in urothelial cell lines led to increased cell migration and epithelial-mesenchymal transition [12]. More recently, Fujii et al. conducted a comprehensive molecular study of 198 UTUC patients through whole-exome sequencing (WES), single nucleotide polymorphism (SNP) array, RNA sequencing, and methylation analysis [37]. They reported 5 molecular subtypes (hypermutated (5.5%), TP53/MDM2 (37.7%), RAS (15.1%), FGFR3 (35.2%) and triple-negative (6.5%)) that correlates with clinicopathological features [37]. Indeed, patients classified in TP53/MDM2 and triple-negative subgroups had the worse disease-specific survival. Moreover, similar to previous studies, they found a majority of luminal-like tumors (71.5%) [37]. They also confirmed a different mutational profile between tumors arising in ureters versus renal pelvis, suggesting a distinct carcinogenesis mechanism depending on location in the urothelium [37]. They also sequenced urinary sediment-derived DNA and found 82.2% (95% CI, 71.5−90.2%) of sensitivity and 100% (95% CI, 81.5−100%) of specificity of this approach for UTUC diagnosis [37].

## 3. The Immune Microenvironment of UTUC

The immune landscape of tumors is an important determinant of the host’s antitumoral response and clinical outcomes [38]. According to the results of molecular profiling studies, it is well established that sporadic UTUC displays a high rate of *FGFR3* mutations (40–80%) [4,5,11,13,22]. Moreover, it has also been shown that upper tract tumors with *FGFR3*-alterations express lower CD8 T-cell gene signatures [11,39]. In addition, *FGFR3* upregulation has been shown to be important in shaping the observed T-cell depleted phenotype [11]. Indeed, genes related to IFNG pathway, such as *BTS2* and *IRF9,* were found downregulated in T-cell depleted cluster and the use of *FGFR3* inhibitors correlated with upregulation of these genes [11]. However, how *FGFR3* signaling mediates IFNG related genes and a T-cell depleted microenvironment has to be determined. Recently, Rose et al. found that upregulated *FGFR3* signaling significantly correlates with upregulated PPARG gene signatures [40]. They suggested it as a potential mechanism given that the upregulation of PPARG signaling appears to suppress pro-inflammatory cytokine signaling and correlates with a non-T cell-inflamed phenotype [40].

Therefore, sporadic UTUC with *FGFR3* mutations might be considered immune cold tumors. Consistent with previous studies, we recently observed similar findings. In addition, we found that *FGFR3* mutated UTUC were hypomethylated compared to *FGFR3* wild-type tumors, suggesting crosstalk between genetic and epigenetic phenotypes of these tumors [12]. Notably, FGFR3 wild-type tumors harbored a high rate of SWI/SNF genetic tumor alterations and were associated with a higher level of tumor infiltrated lymphocytes (TILs) [12]. Further studies are needed to fully understand these observations at the mechanistic level allowing us to elucidate better how the genetic alterations of the cancer cells might shape the immune contexture. Importantly, UTUC developed in a Lynch syndrome context because of an MMR loss of function could be considered immune hot tumors [21].

Finally, correlations were observed between PD-L1 positivity on tumoral cells and the worse outcomes in UTUC patients, although PD-L1 expression rate varied between 20−25% depending on the study and the cutoff used [41,42].

## 4. Current Systemic Management of UTUC

### 4.1. Localized Disease

The gold standard treatment for high-risk disease, defined by at least one of the following criteria: hydronephrosis, tumor size ≥ 2 cm, high-grade cytology, high-grade on biopsy, multifocal disease, previous radical cystectomy for bladder cancer, and variant histology [1], the standard is radical nephro-ureterectomy (RNU) with bladder cuff excision associated with lymph node dissection, according to the European Association of Urology (EAU) guidelines [1]. Kidney-sparing surgery could be proposed for patients with low-risk disease or even in patients with high-risk tumors located in the distal ureter to reduce morbidity. This strategy could allow optimal adjuvant chemotherapy administration by preserving kidney function [1]. For locally advanced UTUC, adjuvant chemotherapy with platinum-gemcitabine combination chemotherapy is recommended.

Indeed, the POUT trial, a multi-center randomized controlled trial including 261 patients, has reported a significant improvement in disease-free survival (DFS) (hazard ratio (HR): 0.45, 95% CI 0.30–0.68; *p* = 0.0001) at a median follow-up of 30.3 months (IQR 18.0–47.5) with adjuvant platinum-gemcitabine combination chemotherapy beginning within 90 days following nephroureterectomy [43]. The recently updated results of this trial, with a median follow-up of 48.1 months (IQR: 36.0–60.1), confirmed the chemotherapy benefit in DFS (HR: 0.50, 95% CI: 0.34–0.75; *p* = 0.001) [44]. There was no detrimental long-term toxicity observed. Furthermore, no statistically significant improvement in OS was observed [44]. Unfortunately, the optimal administration of cisplatin was not feasible for some patients due to the surgical impact on renal function [45,46]. Therefore, neoadjuvant chemotherapy was proposed, with interesting results in terms of pathological downstaging and survival [47]. Moreover, there is a benefit in terms of pathological response, disease recurrence, and mortality rate compared to RNU alone, even if no randomized controlled trials have been published yet [48]. The use of ICIs in the peri-operative setting is not yet a standard of care in UTUC management [1]. Recently published data about the use of ICI in this setting are discussed below.

### 4.2. Metastatic Disease

The management of the metastatic disease should be multimodal, with the use of local therapy in the case of oligometastatic disease, even in the absence of a randomized controlled trial [1,49]. RNU could also be performed in the oligometastatic situation, although providing only a small benefit [50,51,52,53], or in the palliative setting to manage symptomatic disease [1,54].

In the metastatic setting, systemic chemotherapy is extrapolated from data collected in metastatic bladder UC, since few data are available for advanced UTUC [1]. Indeed, cisplatin-based regimens, such as MVAC (methotrexate, vinblastine, doxorubicin, and cisplatin), or GC (gemcitabine and cisplatin) remained standard of care in first-line. Furthermore, a recent retrospective study found that the location of the primary tumor (upper vs. lower tract) did not change the progression-free survival (PFS) and overall survival (OS) in patients treated with platinum-based chemotherapy for metastatic UC [52]. Immune checkpoint inhibitors are also part of the therapeutic armamentarium.

Recently, avelumab as first-line maintenance therapy for platinum-based chemotherapy responder or stable disease became the standard of care [55]. The JAVELIN-100 trial is a randomized controlled phase 3 trial comparing avelumab versus placebo as maintenance therapy after platinum-based chemotherapy in first-line for patients with metastatic UC. A total of 700 patients were randomized, 30.3% (*n* = 106/350) versus 23.1% (*n* = 81/350) of UTUC patients were, respectively, in the avelumab and placebo arm. Median overall survival was significantly longer in the avelumab arm, 21.4 (18.9–26.1) versus 14.3 (12.9–17.9) hazard ratio for death 0.69 (0.56–0.86) *p* = 0.001 [55]. Based on subgroup analysis, the second-line systemic standard of care includes ICI, such as pembrolizumab. Indeed, in the phase 3 trial KEYNOTE-045, 542 UC patients were randomly assigned between pembrolizumab or chemotherapy chosen by the investigator. There was 14.1% (*n* = 38/270) UTUC patients in the pembrolizumab arm versus 13.6% (*n* = 37/272) in the chemotherapy arm. In the total population, median overall survival was 10.3 months (95% CI, 8.0 to 11.8) versus 7.4 months (95% CI, 6.1 to 8.3) in the chemotherapy group (HR, 0.73; 95% CI, 0.59 to 0.91; *p* = 0.002) [56,57]. Nevertheless, based on subgroup analysis, a systemic chemotherapy regimen with a single agent such as paclitaxel, docetaxel, or vinflunine could still be proposed [58].

## 5. Immune Checkpoint Inhibition in UTUC

Given their relative rarity, there are no studies specifically focusing on UTUC. Therefore, data related to ICI efficacy are extracted from a larger cohort of patients with UC that included a small subgroup of UTUC.

### 5.1. Immune Checkpoint Inhibitors in the Perioperative Setting

In the adjuvant setting, ICI-based therapy has been widely tested since the role of adjuvant treatment in high-risk muscle-invasive urothelial carcinoma after radical surgery was not clear.

Since the first promising results of adjuvant pembrolizumab in the management of UC [59,60], several studies have now included UTUC patients in ICI-based adjuvant treatment (Table 1).

The IMvigor 010 study enrolled 809 high-risk UC patients to be randomized between adjuvant atezolizumab versus placebo. There were only 7% of UTUC patients in the atezolizumab arm as compared to 6% in the placebo arm (Table 1). There was no statistical difference in terms of median disease-free survival, 19.4 months (95% CI 15.9–24.8) with atezolizumab and 16.6 months (11.2–24.8) with observation (stratified hazard ratio 0.89 [95% CI 0.74–1.08]; *p* = 0.24) [61]. More recently, the data of the Checkmate 274 trial were reported. It was a phase 3 trial, including 709 patients randomized between adjuvant nivolumab versus placebo. A total of 21% of enrolled patients were patients with UTUC (Table 1). However, based on the results of the POUT trial, the inclusion of UTUC patients was prematurely interrupted. In the intention-to-treat population, median disease-free survival was 20.8 months (95% CI, 16.5 to 27.6) with nivolumab and 10.8 months (95% CI, 8.3 to 13.9) with placebo; (HR, 0.70; 98.22% CI, 0.55 to 0.90; *p* < 0.001). For the UTUC subgroup, the HR for disease recurrence or death were 1.23 (CI 95% 0.67–2.23) and 1.56 (CI 95% 0.7–3.48) for UTUC arising in renal pelvis and ureter, respectively. The percentage of patients was 74.5% and 55.7%, respectively (hazard ratio, 0.55; 98.72% CI, 0.35 to 0.85; *p* < 0.001), for those expressing PD-L1 more than 1% [62]. Moreover, several neoadjuvant trials combining chemotherapy with ICI are actively recruiting, but available data are currently limited [63,64,65].

### 5.2. Immune Checkpoint Inhibitors in the Metastatic Setting

In the metastatic setting, ICI are widely used in the management of UC (Table 2). Indeed, avelumab as maintenance therapy after platinum-based chemotherapy is currently the standard of care according to the results of the JAVELIN-100 trial [55]. For cisplatin-ineligible UC patients, based on phase 2 trials IMvigor 210 and KEYNOTE 052 provided interesting results for the use of ICI in this frail population (Table 2) [66,67]. The overall response rate for UTUC patients was 39% with atezolizumab [66] and 22% with pembrolizumab in monotherapy in this setting [67].

The IMvigor 130 trial, a randomized phase 3 trial, showed significant PFS improvement of the addition of atezolizumab to platinum-based chemotherapy [68] (Table 2). Specific outcomes of UTUC patients were not assessed. However, unfortunately, atezolizumab in monotherapy failed to improve overall survival compared to chemotherapy in pretreated metastatic UC [70]. In the same manner, the addition of pembrolizumab to first-line platinum-based chemotherapy was not associated with a survival benefit compared to chemotherapy alone (Table 2) [69].

In the second or later line, atezolizumab, durvalumab, avelumab, nivolumab, and pembrolizumab have been demonstrated safe and efficient in platinum pretreated UC population. However, UTUC patients’ data remained scarce (Table 2).

## 6. Perspectives in UTUC Management

According to the recent advances in the molecular characterization of UTUC, there is a rationale to develop new treatment combinations. Indeed, given the high prevalence of *FGFR3* mutations and their association with a T-cell depleted phenotype in UTUC, there is a rationale for combining ICI with *FGFR3* inhibitors (Figure 1). Erdafitinib, a pan-FGFR inhibitor, is now approved based on the results of the phase 2 trial in metastatic bladder cancer, with a 40% of response rate in patients with *FGFR* actionable alterations [71]. Moreover, Ding et al. reported the case of a 67 years old metastatic, chemo-refractory UTUC’s patient having a dramatic response to pembrolizumab in association with erdafitinib [72]. However, reliable response biomarkers are still needed to improve precision medicine in urothelial carcinoma. The ongoing trials assessing immune checkpoint inhibitors-based combinations therapies in UTUC metastatic setting are reported in Table 3. They often include backbone ICI in combination with chemotherapy, antibody-drug conjugates, and tyrosine kinase inhibitors.

## 7. Conclusions

ICI inhibitors are now widely used in daily practice to treat urothelial carcinoma patients. Based on the recent advancement in the comprehension of the molecular biology of UTUC and the differences between bladder UC and UTUC, further studies focused on UTUC patients are needed to personalize the therapeutic approach and find new treatment combinations.

## Figures and Tables

**Table 1:** Adjuvant and neoadjuvant systemic treatment for UTUC patients.

Trial	Drug	Study Design	Line	Overall pts *n*, UTUC pts *n*. (%)	Outcomes (Primary Endpoint)
IMvigor 010 [61]	Atezolizumab	Phase 3 RCT	Adjuvant	809; 54 (6.7%)	Median disease-free survival, 19.4 months (95% CI 15.9–24.8)
Checkmate 274 [62]	Nivolumab	Phase 3 RCT	Adjuvant	709; 149 (21%)	Median disease-free survival 20.8 months (95% confidence interval [CI], 16.5 to 27.6)
NCT02690558 [63]	Cisplatin, gemcitabine, pembrolizumab	Phase 2	Neoadjuvant	39; na	pCR:36%
POUT [43]	Cisplatin or carboplatin + gemcitabine	Phase 3 RCT	Adjuvant	261	Disease-free survival (hazard ratio 0.45, 95% CI 0.30–0.68; *p* = 0.0001)

Abbreviations: RCT: randomized controlled trial, pts: patients; na: non available; pCR: pathologic complete response.

**Table 2:** Studies assessing ICI in patients with locally advanced or metastatic UC (only trials reporting data of UTUC patients were selected).

Trial	Drug/Control Arm	Study Design	Line	Overall pts *n*, UTUC pts *n*. (%)	Outcomes (Primary Endpoint)
JAVELIN-100 [55]	Avelumab/BSC	Phase 3 RCT	1L	700, 187 (27%)	median OS: 21.4 months vs. 14.3 months; hazard ratio for death, 0.69; 95% confidence interval [CI], 0.56 to 0.86; *p* = 0.001
KEYNOTE 052 [67]	Pembrolizumab	Phase 2	1L	370, 69 (19%)	ORR: 24%, 95% CI 20–29)
IMvigor 130 [68]	Atezolizumab + platinum-based chemotherapy (A)/Atezolizumab (B)/Platinum-based chemotherapy	Phase 3 RCT	1L	1213, 312 (26%)	median PFS: 8.2 months (95% CI 6.5–8.3) in group A and 6.3 months (6.2–7.0) in group C (stratified hazard ratio [HR] 0.82, 95% CI 0.70–0.96; one-sided *p* = 0.007). median OS: 16.0 months (13.9–18.9) in group A and 13.4 months (12.0–15.2) in group C (0.83, 0.69–1.00; one-sided *p* = 0.027). Median overall survival was 15.7 months (13.1–17.8) for group B and 13.1 months (11.7–15.1) for group C (1.02, 0.83–1.24)
KEYNOTE 361 [69]	Cisplatin or Carboplatin + Gemcitabine + Pembrolizumab/Pembrolizumab/Cisplatin or Carboplatin + Gemcitabine	Phase 3 RCT	1L	1010, 211 (21%)	median OS: 17·0 months (14.5–19.5) in the pembrolizumab plus chemotherapy group versus 14.3 months (12.3–16.7) in the chemotherapy group (0.86, 0.72–1.02; *p* = 0.0407) median PFS: 8.3 months (95% CI 7.5–8.5) in the pembrolizumab plus chemotherapy group versus 7.1 months (6.4–7.9) in the chemotherapy group (hazard ratio [HR] 0.78, 95% CI 0.65–0.93; *p* = 0.0033)
KEYNOTE-045 [56]	Pembrolizumab/Paclitaxel or Docetaxel or Vinflunine	Phase 3 RCT	2L	748, 75 (10%)	median OS: 10.3 months (95% CI 8.0 to 11.8) vs. 7.4 months (95% CI, 6.1 to 8.3) (hazard ratio for death, 0.73; 95% CI, 0.59 to 0.91; *p* = 0.002) median PFS: 2.1 months (95% CI, 2.0 to 2.2) vs. 3.3 months (95% CI, 2.3 to 3.5) (HR 0.98; 95% CI, 0.81 to 1.19; *p* = 0.42)
IMvigor 211 [70]	Atezolizumab/Paclitaxel or Docetaxel or Vinflunine	Phase 3 RCT	2L	931, 236 (25%)	median OS: 11.1 (95% CI 8.6–15.5) vs. 10.6 months (95% CI 8.4–12.2) *p* = 0.41
IMvigor 210 [66]	Atezolizumab	Phase 2	2L	119, 33 (28%)	ORR: 23% (95% CI 16–31)

Abbreviations: BSC: best supportive care; RCT: randomized controlled trial; OS: overall survival; PFS: progression free survival; ORR: objective response rate.

**Table 3:** Ongoing trials assessing immune checkpoint inhibitors-based combinations therapies in the metastatic setting.

Trial Identification	Drugs	Comparative Arm	Administration	Study Design	Line	Primary Endpoint
NCT03513952	Atezolizumab/CYT107	Atezolizumab	IV	Phase 2	≥2	ORR
NCT03237780	Atezolizumab/eribulin	Eribulin	IV	Phase 2	>2	ORR
NCT02496208	Cabozantinib/Nivolumab ± Ipilimumab	NA	PO/IV	Phase 1	>1	RP2D/safety
NCT04940299	Tocilizumab/Ipilimumab/Nivolumab	NA	IV	Phase 2	1	Safety/DLT
NCT03606174	Sitravatinib/Nivolumab and Sitravatinib/Pembrolizumab/Enfortumab vedotin	NA	PO/IV and PO/IV/IV	Phase 2	1, ≥2	ORR
NCT04602078	Atezolizumab/Gemcitabine/Cisplatin	NA	IV	Phase 2	1	ORR
